# Prevalence of hemorrhagic fever with renal syndrome in Yiyuan County, China, 2005–2014

**DOI:** 10.1186/s12879-016-1404-7

**Published:** 2016-02-06

**Authors:** Tao Wang, Jie Liu, Yunping Zhou, Feng Cui, Zhenshui Huang, Ling Wang, Shenyong Zhai

**Affiliations:** Department of Infectious Disease Control and Prevention, Zibo Center for Disease Control and Prevention, Zibo, Shandong Province P. R. China

**Keywords:** Hemorrhagic fever with renal syndrome, Autoregressive integrated moving average model, Prevalence

## Abstract

**Background:**

Hemorrhagic fever with renal syndrome (HFRS) is highly endemic in mainland China, where human cases account for 90 % of the total global cases. Yiyuan County is one of the most serious affected areas in China. Therefore, there is an urgent need for monitoring and predicting HFRS incidence in Yiyuan to make the control of HFRS more effective.

**Methods:**

The study was based on the reported cases of HFRS from the National Notifiable Disease Surveillance System. The demographic and spatial distributions of HFRS in Yiyuan were established. Then we fit autoregressive integrated moving average (ARIMA) models and predict the HFRS epidemic trend.

**Results:**

There were 362 cases reported in Yiyuan during the 10-year study period. The human infections in the fall and winter reflected a seasonal characteristic pattern of Hantaan virus (HTNV) transmission. The best model was ARIMA (2, 1, 1) × (0, 1, 1)_12_ (AIC value 516.86) with a high validity.

**Conclusion:**

The ARIMA model fits the fluctuations in HFRS frequency and it can be used for future forecasting when applied to HFRS prevention and control.

## Background

Hemorrhagic fever with renal syndrome (HFRS), a rodent-borne disease caused by hantaviruses (family Bunyaviridae), is characterized by fever, acute renal dysfunction, and hemorrhage manifestations [1–3]. HFRS was first recognized in northeastern China in 1931 and has been prevalent in many other parts of China since 1955. At present, it is highly endemic in mainland China accounting for 90 % of the total cases reported in the world [4–7]. In response to the spread of HFRS in China, the Chinese Center for Disease Control and Prevention (CDC) established the National Notifiable Disease Surveillance System in 2004, which made the surveillance data for HFRS more accurate and comprehensive. A better understanding of the spatial distribution patterns and social demographic distribution characteristics of HFRS would help to identify areas and populations at high risk. Early warnings are also essential for controlling or reducing the risk of outbreaks [8], epidemic modeling and forecasting can be essential tools to prevent and control HFRS [9]. In epidemiology, Autoregressive integrated moving average (ARIMA) models have been successfully applied to predict the incidence of infectious diseases, such as HIV [10], influenza [11], malaria incidence [12], and other infectious diseases [13–15].

This study aimed to establish the current situation of endemic HFRS in Yiyuan, and characterize its spatio-temporal distribution and demographic distribution characteristics. Furthermore, we fit ARIMA models and predict the HFRS epidemic trend by using SAS version 9.2 (SAS Institute, Cary, NC,USA). Our study was based on HFRS epidemic data from Yiyuan County, China, where it could provide a basis for HFRS prevention and control.

## Methods

### Study area

The study site is located in Yiyuan County (latitude 35°55′ ~ 36°23′ N and longitude 117°54′ ~ 118°31′ E), in the central part of Shandong province. Monthly HFRS cases reported during 2005–2014 were provided by Zibo CDC, and we were permitted to use the data. In China, HFRS is a nationally notifiable disease and hospital physicians must report every case of HFRS to the local health authority within 24 h.

### Ethics statement

The ethical approval was given by Ethics Review Committee of the Zibo Center for Disease Control and Prevention, and the study was conducted in compliance with the principles of the Declaration of Helsinki. Written informed consents for the use of their clinical samples were obtained from the patients and all analyzed data were anonymized. Besides, consents of participants who were under 16 years have been obtained from their parents/guardians.

### Demographic distribution analysis

The demographic distribution characteristics including age, sex and occupation distribution of HFRS cases from 2005 to 2014 in Yiyuan County were analyzed according to surveillance data. All HFRS cases were geo-coded and matched to the town-level layers of polygon and point by administrative code using the software ArcGIS9.3 (ESRI Inc., Redlands, CA, USA). To alleviate variations of incidence in small populations and areas, annualized average incidence of HFRS per 100 000 at each town over the 10 year-period were calculated. Furthermore, annualized average incidences and the proportion of monthly average incidence for each town were mapped in gradient colors and pie charts, respectively. To approximately distinguish the dominant hantaviruses, we divided a year into three periods according to the seasonal distribution of the HFRS cases: March to June (in spring and early summer), July to August (in summer), and September to February (in autumn and winter).

### Time-series analysis

ARIMA models are the most commonly used time series prediction models [10]. We constructed ARIMA models for monthly HFRS incidence in Yiyuan from 2005 to 2014. ARIMA was designed to deal with highly seasonal data [16]. An ARIMA (p, d, q) model comprises three types of parameters [9, 16, 17]: the autoregressive parameters (p), number of differencing passes (d), and moving average parameters (q). The multiplicative seasonal ARIMA (p, d, q) × (P, D, Q)s model is an extension of the ARIMA method to time series in which a pattern repeats seasonally over time [15, 16, 18]. Analogous to the simple ARIMA parameters, the seasonal parameters are: seasonal autoregressive (P), seasonal differencing (D), and seasonal moving average parameters (Q). The length of the seasonal period is represented by s. For example, the incidence of infectious disease varies in the annual cycle, so s = 12 in the present study.

We used the Box-Jenkins strategy to construct models. The ARIMA model procedure consists of three iterative steps [15, 17, 18]: identification, estimation, and diagnostic checking. Prior to fitting the ARIMA model, an appropriate difference of the series is usually performed to make the series stationary. Identification is the process of determining seasonal and non-seasonal orders using the autocorrelation functions (ACF) and partial autocorrelation functions (PACF) of the transformed data. Parameters in the ARIMA model(s) are estimated with the conditional least squares method after the identification step. At the diagnosis stage, the adequacy of the established model for the series is verified by employing white noise tests to check whether the residuals are independent and normally distributed. It is possible that several ARIMA models may be identified, and the selection of an optimum model is necessary. Such selection of models is usually based on the Akaike Information Criterion (AIC) and Schwartz Bayesian Criterion (SBC). Smaller AIC values indicate a better model, and the SBC considers the residual error, which is based on AIC. The lowest SBC value with a *P* value less than 0.05 was considered to be the best model [19]. In addition, to check the accuracy of each model, root mean square error (RMSE) between the number of observed and fitted HFRS infections from 2005 to 2014 were calculated. A lower RMSE value indicates a better fit of the data. Finally, the fitted ARIMA model was used for short-term forecasting of the monthly HFRS incidence between January and December 2014. All analyses were performed using SAS 9.2 with a significant level of *p* < 0.05.

## Results

### Descriptive analysis of HFRS in Yiyuan County

**Fig. 1:**
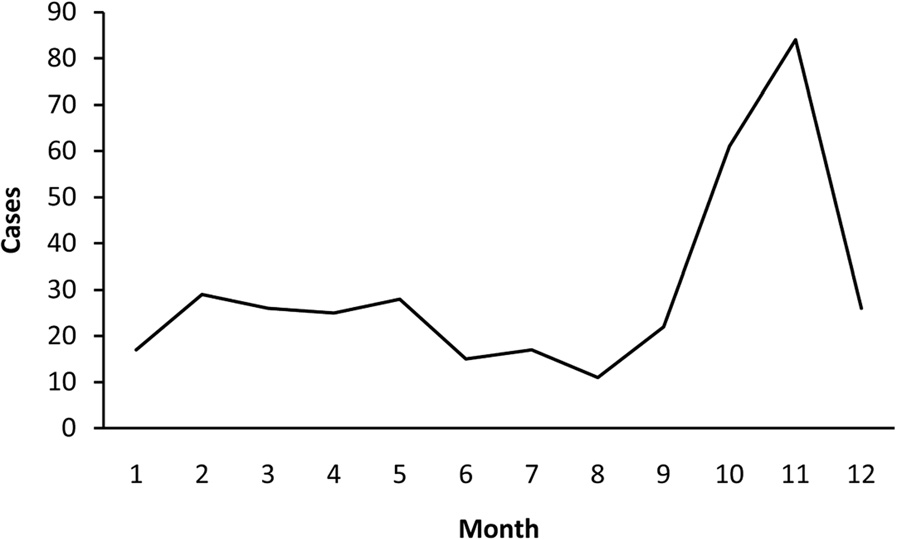
Monthly distribution of HFRS cases, 2005–2014

A total of 362 cases were reported in Yiyuan County during the 10-year study period. Of these, 65 % were male and 35 % were female, with the sex ratio (male vs. female) 1.85. Among these patients, 1 % were in children ≤14 years of age, 88 % were in persons 15–64 years of age, and 11 % were in persons ≥65 years of age. Regarding to occupation, 89 % of HFRS patients were farmers, 5 % were workers (mainly forestry workers, builders), and followed by students which accounted for 4 %. Poor housing conditions and high rodent density in rural areas seem to be responsible for most HFRS epidemics. The monthly distribution of HFRS cases was shown in Fig. 1, which indicated that the occurrence of HFRS presented significant seasonality.

**Fig. 2:**
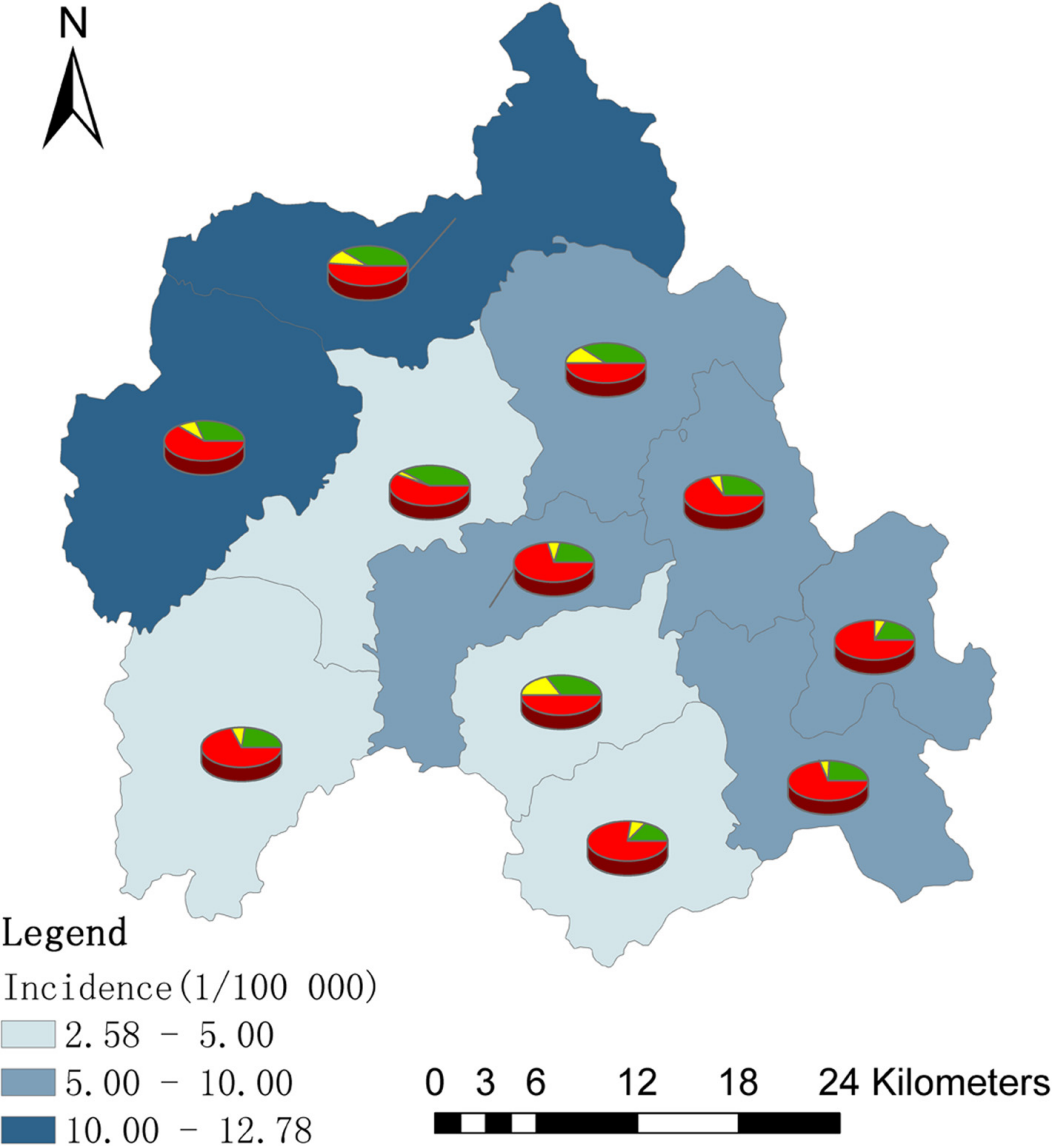
The spatial distribution of HFRS incidence and their proportion of monthly incidence in each town. The background of map with color gradient presents the annual incidence of HFRS, and pie graphs display the proportion of monthly incidence for each town. Green color indicates the proportion of average monthly incidence from March to June (in spring and early summer), yellow is the proportion of average monthly incidence from July to August (in summer), and the red represents the proportion of average monthly incidence from September to February (in autumn and winter)

Figure 2 showed annualized average incidence and the proportion of monthly average incidence for each town. Annualized average incidence at the town-level ranged from 2.58 to 12.78 per 100 000. Among the total 11 towns in Yiyuan, 7 towns were medium-endemic with an incidence between 5 and 15 per 100 000, an epidemic peak in the fall-winter season was mapped by the red color in the pies.

### Model identification

**Fig. 3:**
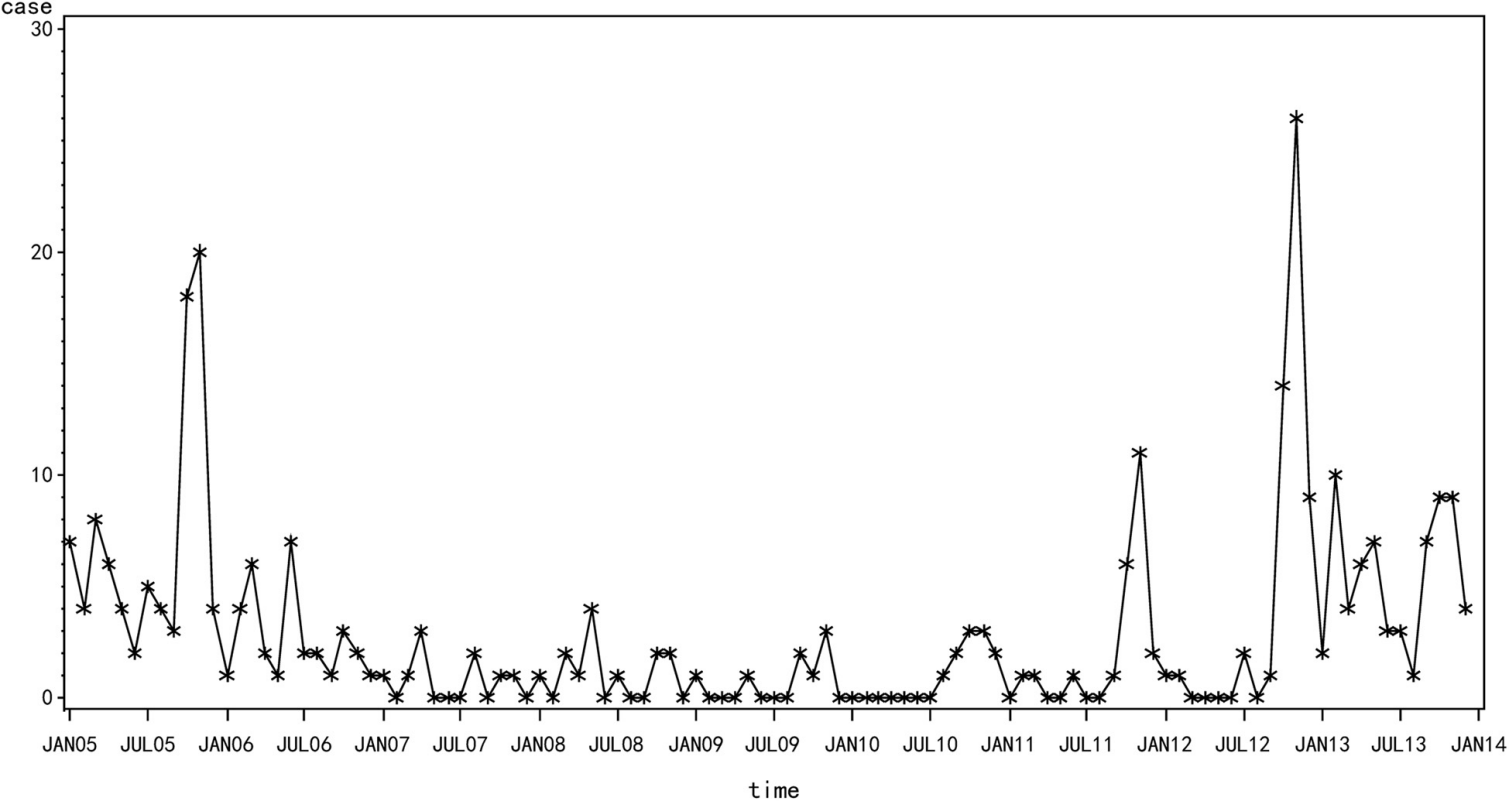
The cases of HFRS in Yiyuan County from 2005 to 2013

**Fig. 4:**
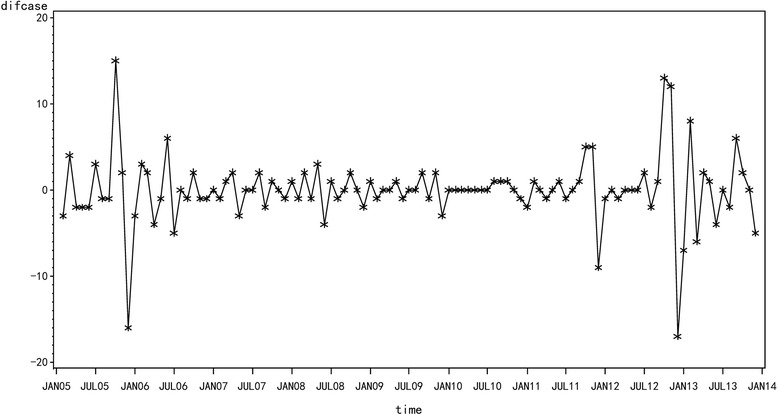
1-order difference of HFRS in Yiyuan County from 2005 to 2013

**Fig. 5:**
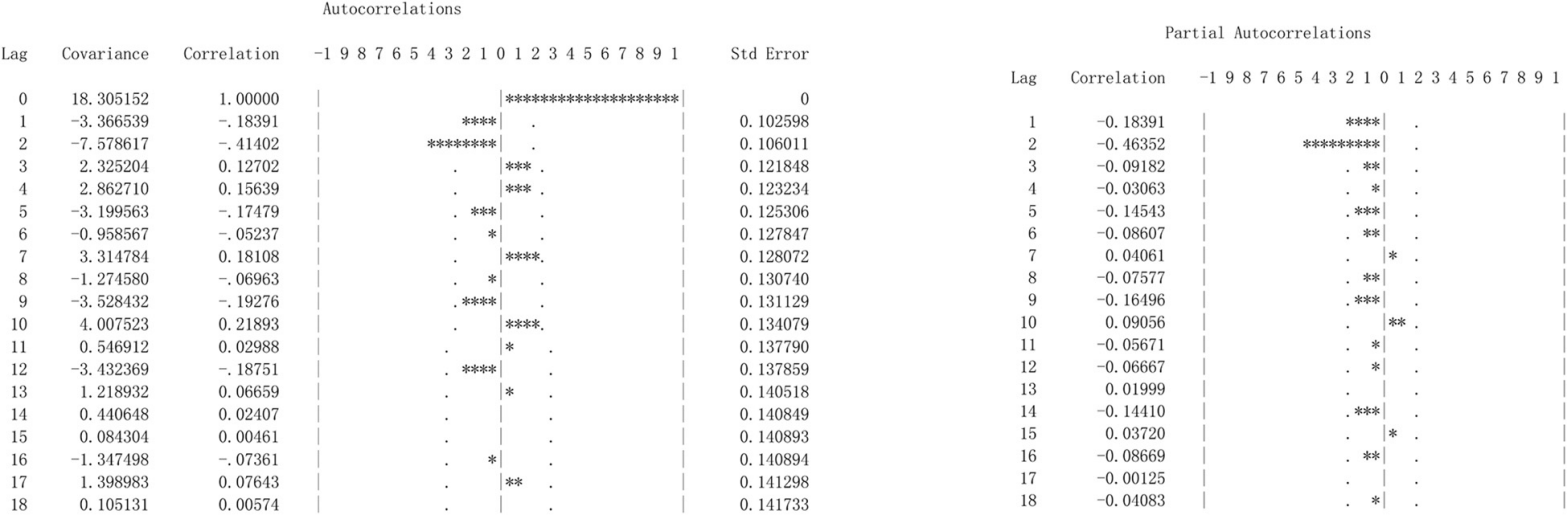
Autocorrelation function and partial autocorrelation function of 1-order difference. Dotted line: 95 % confidence intervals

Time series data for HFRS covering 2005–2013 in Yiyuan were used as the training set and monthly data for 2014 were used as the test set (Fig. 3). The sequence diagrams and the seasonal characteristics of HFRS incidence indicated that the data series had a seasonal cycle every 12 months. On the basis of these characteristics, we eliminated the effect of seasonal trends by taking 1-order trend difference and 1-order seasonal difference. The transformed series showed far less dispersion than original series (Fig. 4). Plausible models, i.e., (the ARIMA (2, 1, 1) × (0, 1, 0)_12_, ARIMA (2, 1, 0) × (0, 1, 1)_12_, ARIMA (1, 1, 1) × (1, 1, 1)_12_ and ARIMA (2, 1, 1) × (0, 1, 1)_12_), were identified on the basis of autocorrelation functions (ACF) and partial autocorrelation functions (PACF) (Fig. 5), and were used for further analysis.

### Parameter estimation and model testing

On the basis of parameter estimation and goodness of fit test statistics (Tables 1 and 2), we confirmed that the best model was ARIMA (2, 1, 1) × (0, 1, 1)_12_. The goodness-of-fit analysis showed that there was no significant autocorrelation between residuals at different lags.

**Table 1:** Parameter estimation for plausible ARIMA models^a^

Parameter	ARIMA (2, 1, 1) × (0, 1, 0)_12_			ARIMA (2, 1, 0) × (0, 1, 1)_12_			ARIMA (1, 1,1) × (1, 1, 1)_12_			ARIMA (2, 1, 1) × (0, 1, 1)_12_		
	B	t	*P*	B	t	*P*	B	t	*P*	B	t	*P*
AR1,1	−0.479	−4.510	<0.001	−0.463	−4.740	<0.001	0.422	3.070	0.003	−0.458	−4.460	<0.001
AR2,1							0.449	1.800	0.075			
MA1,1	0.308	3.020	0.003				0.873	11.100	<0.001	0.275	2.650	0.009
MA2,1				0.375	3.580	<0.001	0.743	3.850	<0.001	0.314	2.910	0.005

^a^ AR1,1 = autoregressive parameter; AR2,1 = seasonal autoregressive parameter; MA1,1 = moving average parameter; MA2,1 = seasonal moving average parameter

**Table 2:** Goodness of fit statistics for plausible ARIMA models^a^

Models	AIC	SBC
ARIMA (2, 1, 1) × (0, 1, 0)_12_	519.854	524.962
ARIMA (2, 1, 0) × (0, 1, 1)_12_	520.812	525.920
ARIMA (1, 1, 1) × (1, 1, 1)_12_	522.897	533.113
ARIMA (2, 1, 1) × (0, 1, 1)_12_	516.855	524.517

^a^ AIC = Akaike information criterion; SBC = Schwarz Bayesian criterion

### Forecast analysis

**Fig. 6:**
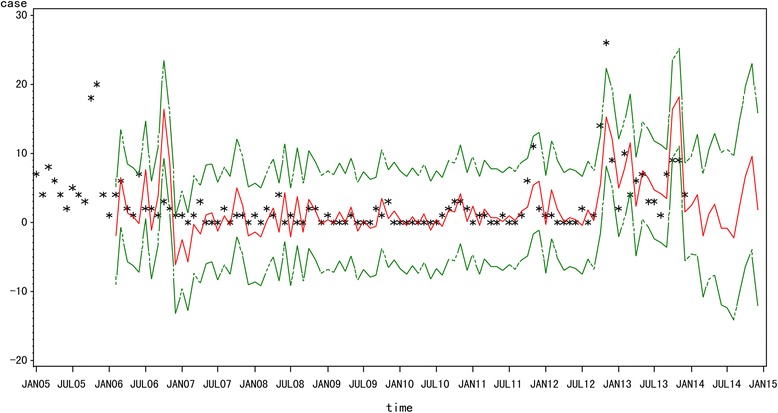
Fitted, predicted and actual incidence of HFRS in Yiyuan County from 2005 to 2014. Black star: actual HFRS incidence; red solid line: ARIMA (2, 1, 1) × (0, 1, 1)_12_ model fitted curve; green dashed lines: 95 % confidence intervals of fitted values

We used ARIMA model (2, 1, 1) × (0, 1, 1)_12_ and time series data for 2005–2013 as the training set, and the data from January-December 2014 were used as the test set (Fig. 6, and Table 3). The predicted data for the actual data and the predicted data 95 % confidence limit for 2014 are shown in Table 3. The RMSE value was 3.56. The predicted data and the actual data were not perfectly matched, but the actual data fell within the predicted 95 % confidence interval.

**Table 3:** Comparison of predicted HFRS cases and actual values for Yiyuan, during January-December 2014^a^

No cases	Jan	Feb	Mar	Apr	May	Jun	Jul	Aug	Sep	Oct	Nov	Dec
Actual value	3	9	4	7	11	2	4	1	5	4	7	4
Predictive value	2	4	0	1	3	0	0	0	2	7	9	2
95 % CL of PV	0–10	0–13	0–7	0–11	0–13	0–10	0–11	0–10	0–15	0–20	0–23	0–16

^a^ CL = confidence level; PV = predicted value

## Discussion

In this study, our results demonstrated that the HFRS incidence had been increasing from 2009 to 2014 in Yiyuan County. The proportion of monthly average incidence for each town showed that HFRS in Yiyuan was mainly caused by HTNV. We applied multiplicative seasonal ARIMA (p, d, q) × (P, D, Q)s models to analyze the surveillance data of HFRS in Yiyuan, China. According to the results above, the ARIMA (2, 1, 1) × (0, 1, 1)_12_ model is reliable with a high validity, which can be used to predict the next one year’s HFRS incidence in Yiyuan.

Yiyuan, are mountainous and hilly with numerous *Apodemus agrarius*, and farmers have more chances to be exposed to contaminated urine and feces of infected rodents. Agricultural activities such as sleeping in the fields, irrigating, and working on the farmland during the autumn harvest season might have played a significant role in the occurrence of HFRS [20]. Previous studies reported that the transmission of HTNV through *Apodemus agrarius* peaked in the winter, while *Rattus norvegicus* associated SEOV infections mainly occurred in the spring [21–24]. Thus, the human infections in Yiyuan in the fall and winter reflect a seasonal characteristic pattern of HTNV transmission. The forecast results suggest that the HFRS incidence in China will experience a slight growth in the next one year. A rise in the number of HFRS incidence may also result from an increase in the number and size of natural foci [25], climate change, especially the increase of mean temperature [20, 23]. Therefore, knowledge of HFRS forecasts is necessary to prompt health departments to strengthen surveillance systems and reallocate resources in anticipation of increasing HFRS incidence.

Epidemiological surveillance of communicable diseases is one of the most traditional health-related activities. Time-series analysis of incidence of various infections is extremely useful in developing hypotheses to explain and anticipate the dynamics of the observed phenomena and subsequently in the establishment of a quality control system and reallocation of resources [26]. There are a number of methods applied for time series analysis including ARIMA model [8–13, 16], maximum entropy method (MEM) spectral analysis [27] and the autoregressive conditional heteroscedastic (ARCH) model [28]. The ARIMA model has its advantages in time-series analyses. The secular trend, seasonal variation, and autocorrelation could all be easily controlled by difference, auto-regression, moving average, and seasonal functions without performing complicated transformations or using extra surrogate variables [18]. Once a satisfactory model has been obtained, it can be used to forecast expected numbers of cases for a given number of future time intervals [29].

Besides, the application of GIS, together with time-series analyses in the present study, provides ways to quantify explicit HFRS and to further identify environmental factors responsible for the increasing disease risk. Although analyses are still preliminary, the findings can be helpful for generating hypothesis for further investigation. For example, based on the prediction results, the government can invest more health resources during high-risk periods and decrease it during low-risk periods to improve the cost-effectiveness of interventions and scheduling of resources. It can also be used to evaluate the effectiveness of public health interventions under varying assumptions by comparing actual HFRS incidence with expected incidence.

However, limitations should also be considered in this present study. The RMSE value was 3.56, and the actual data did not match the predicted data of the model perfectly. Due to a lack of time series data on the population densities of rodents, and the influencing factors, it is difficult to further uncover the probable causes and shifts of the characters of HFRS. Future researches are warranted to focus on the risk factors of HFRS to modify the ARIMA model such as rodent population densities, human activities, farming patterns, various socio-economic and environmental factors in Yiyuan.

## Conclusion

In summary, the results of our study provide useful information on the prevailing epidemiological situation of HFRS in Yiyuan. We further confirmed the consensus that ARIMA model is a useful tool in monitoring and predicting changing trends in HFRS. The ARIMA model could be used to optimize HFRS prevention by providing short-term forecasting on the HFRS incidence. To control and prevent HFRS, a comprehensive preventive strategy including public health education and promotion, rodent control, surveillance, and vaccination should be implemented in Yiyuan.

## Abbreviations

ARIMA: Autoregressive integrated moving average
HFRS: Hemorrhagic fever with renal syndrome
HTNV: Hantaan virus
